# The Relationship of Triphasic Waves with Intracranial Pressure as a Possible Prognostic Marker in Traumatic Brain Injury

**DOI:** 10.1155/2017/4742026

**Published:** 2017-11-28

**Authors:** Nakul Katyal, Aarti Sarwal, Pravin George, Biswajit Banik, Christopher R. Newey

**Affiliations:** ^1^Department of Neurology, University of Missouri, 5 Hospital Drive, CE 540, Columbia, MO 65211, USA; ^2^Wake Forest University School of Medicine, Neurology and Critical Care (Anesthesia), Reynolds M, Medical Center Blvd, Winston-Salem, NC 27157, USA; ^3^Cleveland Clinic, Cerebrovascular Center, 9500 Euclid Avenue, Cleveland, OH 44195, USA

## Abstract

**Background:**

Continuous electroencephalography (CEEG) monitoring is used for detection of convulsive and nonconvulsive seizures and assessing the degree of encephalopathy in critically ill patients. A commonly seen encephalopathic pattern on CEEG is generalized periodic discharges with triphasic wave (TW) morphology. The underlying role and prognostic significance of TW in relationship to intracranial pressure (ICP) remain unknown. We present a case highlighting the relationship of TW with ICP in a case with severe traumatic brain injury (TBI).

**Method:**

Case report.

**Results:**

A patient with severe TBI and no underlying metabolic abnormalities was admitted to the neurocritical care unit. TW were seen on CEEG. The TW diminished during episodes of intracranial hypertension but reappeared with reduction of the intracranial pressure.

**Conclusion:**

This study highlights a possible favorable prognostic marker of finding TW in a patient with intracranial hypertension. We have proposed a preliminary understanding of the relationship between TW and intracranial hypertension, which may be helpful in formulating future studies involving larger cohorts.

## 1. Introduction

Continuous electroencephalography (CEEG) monitoring is commonly used in critically ill patients. CEEG can detect convulsive and nonconvulsive seizures and can be used to assess degree of encephalopathy [1–4]. CEEG is tightly linked to cerebral metabolism and, thus, sensitive to changes in cerebral blood flow [1]. Several patterns on CEEG are of diagnostic and prognostic significance in patients with cerebral edema that lie on the ictal-interictal continuum. EEG patterns that correlate with increased intracranial pressure (ICP) include focal slowing of underlying rhythms or global EEG suppression progressing to burst suppression or flat EEG [1]. Another pattern associated with cerebral edema and oftentimes metabolic derangement on the ictal-interictal continuum is generalized periodic discharges (GPDs) with triphasic wave (TW) morphology.

TW are conspicuous complexes that are frequently encountered on CEEG monitoring in patients with encephalopathy [5]. TW have been linked with various diseases such as hypertensive encephalopathy, hypernatremia/hyponatremia, hypoglycemia, hypercalcemia, brain abscesses, septic shock and sepsis-related encephalopathy, postictal states, lithium and baclofen toxicity, stroke, and renal and/or liver disease [5–7]. The underlying pathogenesis of TW is largely unknown. In patients with hepatic encephalopathy, it is believed to be associated with hyperammonemia and N-methyl-D-aspartate activation [7]. This activation ultimately increases cerebral inhibition by increased gamma–aminobutyric acid (GABA) tone and astrocytic and cytotoxic edema [7, 8]. Another theory proposes that it is primarily a disturbance at the thalamic level with changes in the potential field observed on the cortical surface reflected through thalamocortical relays [9]. Importantly, disturbances in thalamocortical relays can be associated with structural or metabolic abnormalities [9]. This theory better explains the finding of TW in various etiologies other than renal or hepatic disturbances.

The occurrence of TW or their relationship to intracranial hypertension as a possible prognostic tool has not been reported. We present a patient with severe traumatic brain injury (TBI) who was noted to have TW on CEEG. The TW diminished during episodes of intracranial hypertension and reappeared once the elevated ICP was treated.

## 2. Case Report

**Figure 1:**
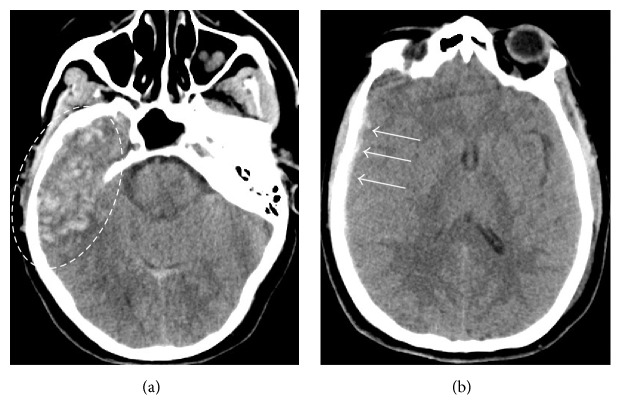
Computed tomography (CT) of the head. CT head showed a right temporal contusion with mixed density and surrounding vasogenic edema (dashed circle; (a)). She also had a small right frontal subdural hemorrhage (arrows; (b)).

**Figure 2:**
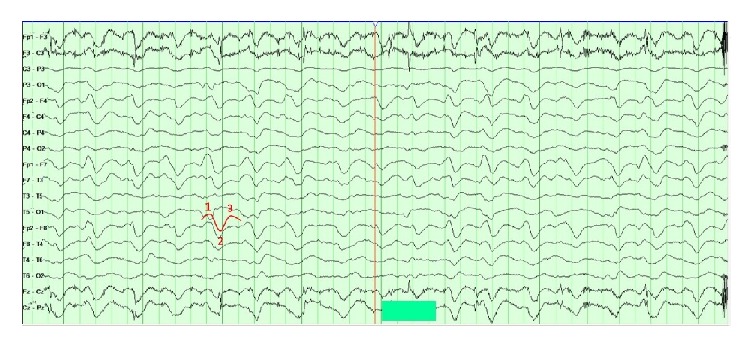
Continuous electroencephalography (CEEG). CEEG shows generalized slowing with triphasic waves. The three phases of the wave that give the classic triphasic morphology are highlighted. Intracranial pressure at the time of this CEEG was 6 mmHg.

**Figure 3:**
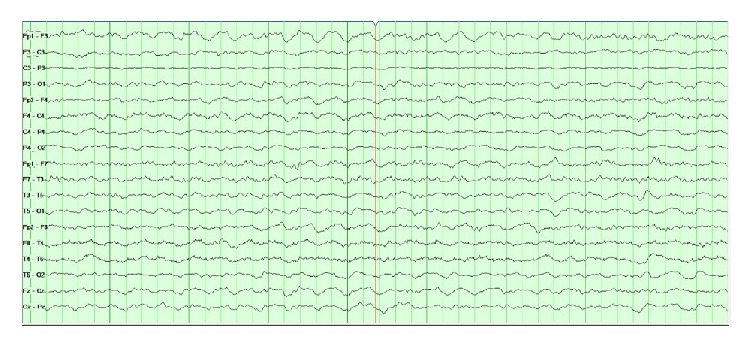
Continuous electroencephalography (CEEG). CEEG shows generalized slowing with attenuation of triphasic waves. Intracranial pressure at the time of this CEEG was 50 mmHg.

A 51-year-old female with a history of hypertension and atrial fibrillation taking warfarin presented to the emergency department after sustaining a ground level fall. Her Glasgow coma scale (GCS) score was 7 (E: 2/V: 1/M: 4). She was intubated for airway protection. Her neurological exam was significant for withdrawal from painful stimulus, bilaterally reactive pupils, brisk deep tendon reflexes (DTR), and bilateral upgoing toes. Computed tomography (CT) head showed a right temporal contusion with surrounding vasogenic edema and a small right frontal subdural hemorrhage (Figure 1). Computed tomography angiography (CTA) was negative for any vascular abnormality. Her international normalized ratio (INR) was 1.8. The coagulopathy was rapidly reversed with 4-factor prothrombin complex concentrate (25 U/kg). She was admitted to the neurosciences intensive care unit. An external ventricular device was placed with opening ICP of 14 mmHg. Overnight, the ICP increased to 25 mmHg and remained between 18 and 22 mmHg for the next 24 to 48 hours. She was treated with osmotherapy (i.e., mannitol and hypertonic saline) and propofol sedation for elevated intracranial pressure (ICP). Her CEEG showed moderate diffuse background slowing and excessive beta and spindle activity likely from sedation. Her elevated ICP responded to cerebrospinal fluid (CSF) drainage, osmolar therapy, and sedation. 72 hours after admission, her ICP became refractory to these treatments, and she required pentobarbital coma which was titrated to burst suppression. Her ICP was controlled between 7 and 20 mmHg. Repeat CT head showed increased vasogenic edema with no new hemorrhages. Over the next few days, pentobarbital was weaned and transitioned to propofol for sedation. CEEG on propofol now showed diffuse slowing and generalized periodic discharges with TW (Figure 2). She then developed paroxysmal episodes of intracranial hypertension with ICP ranging up to 50 mmHg lasting a few minutes. During these episodes, CEEG showed attenuation of TW (Figure 3). Once her intracranial hypertension was reduced with osmotic therapy and sedation, TW reappeared to the pattern shown in Figure 2. Patient's EEG patterns never showed seizures throughout the entire course of monitoring. She continued to improve clinically and was eventually discharged to rehabilitation center for further therapy.

## 3. Discussion

We report changes on CEEG with fluctuations in ICP in a patient with TBI and no metabolic abnormalities. As ICP increased, the TW became diminished, but returned to normal amplitude with lowering of the ICP. This observation highlights a previously unknown relationship between TW and ICPs and a possible favorable prognostic significance of TW as a marker for improved cortical function in a patient with intracranial hypertension secondary to severe TBI.

On CEEG, TW classically have a surface positive wave, preceded and followed by smaller surface negative deflections, giving them a triphasic appearance [10]. TW are usually diffuse and synchronous with bifrontal predominance [10]. Although triphasic waves are more frequently reported in patients with underlying hepatic encephalopathies, they can be associated with structural and/or metabolic derangement [9]. The underlying pathogenesis is believed to be related to dysfunction of thalamocortical relay neurons [9].

The attenuation of TW during intracranial hypertension, as we described, may be attributed to physiological effects of the increased intracranial pressure. Raised intracranial pressure, despite aggressive management, can lead to compromised neuronal oxygenation and widespread reduction in cerebral flow and perfusion [1, 11]. When cerebral blood flow (CBF) becomes compromised, changes occur in both the metabolic and electrical activity of cortical neurons which may be reflected on EEG as changes in frequency [11–13]. EEG changes start occurring when CBF falls below <30% of the normal value [14]. Faster frequencies disappear first, followed by slower frequencies, and eventually the EEG progresses to complete background suppression [11, 13].

Since triphasic waves may be GABAergic-mediated, they are influenced by sedation. A recent study evaluated the empiric treatment of TW in 64 patients [15]. They defined a positive response as resolution of the EEG pattern and either improvement in encephalopathy or appearance or previously absent normal EEG patterns [15]. They ultimately found that 18.9% of patients who received a benzodiazepine challenge had a positive response [15]. In another study of 10 patients, the addition of benzodiazepines resolved TW in 4 patients [16]. However, unresponsive patients did not improve and drowsy patients worsened [16]. This latter observation highlights the importance of recognizing triphasic waves and at the same time not labelling them as being ictal by their EEG improvement alone following a benzodiazepine challenge [16].

In our study, TW attenuated in the presence of plateau waves. Plateau waves are classically seen as sudden rapid elevation of intracranial pressure to 50 to 100 mmHg for 5 to 20 min [17–19]. After a sustained period of elevation, termination of the wave is characterized by a rapid decrease of intracranial pressure. The onset of the plateau wave has been attributed to a vasodilatory cascade model, which leads to increased cerebral blood volume (CBV), elevated intracranial pressure, and decreased cerebral perfusion pressure (CPP) (19, 20). Termination of the wave has been attributed to a vasoconstriction cascade model which causes decreases in CBV and intracranial pressure along with increase of CPP [19, 20]. This cascade model can explain the attenuation of TW with raised ICP and reappearance with decreasing intracranial pressure.

EEG patterns may have correlations to changes in ICP. These changes may have a clinical role in management of patients who cannot receive invasive intracranial pressure monitoring due to coagulopathy or other factors. Awareness of changes in EEG patterns in various critically ill states may lead to future studies creating algorithmic approaches to using CEEG in managing these patients.

## 4. Conclusion

TW are multifactorial in etiology. We describe the reappearance of TW as being a possible favorable prognostic marker in patients with intracranial hypertension. Evolving TW on CEEG may be a predictor for improving cortical function in patients with elevated ICP. Overall, their role and prognostic significance in relationship with increased ICP warrant further investigation.
